# K-means clustering of outpatient prescription claims for health insureds in Iran

**DOI:** 10.1186/s12889-023-15753-1

**Published:** 2023-04-28

**Authors:** Shekoofeh Sadat Momahhed, Sara Emamgholipour Sefiddashti, Behrouz Minaei, Zahra Shahali

**Affiliations:** 1 Department of Health Management and Economics, School of Public Health, Tehran University of Medical Sciences, Tehran, Iran; 2 School of Computer Engineering, Iran University of Science and Technology, Tehran, Iran; 3 National Center for Health Insurance Research (Iran Health Insurance Organization), Tehran, Iran

**Keywords:** Health insurance, k-means clustering, Prescription claims, Medication cost, Risk class

## Abstract

**Objective:**

The segmentation of consumers based on their behavior and needs is the most crucial action of the health insurance organization. This study's objective is to cluster Iranian health insureds according to their demographics and data on outpatient prescriptions.

**Setting:**

The population in this study corresponded to the research sample. The Health Insurance Organization's outpatient claims were registered consecutively in 2016, 2017, 2018, and 2019 were clustered.

**Design:**

The k-means clustering algorithm was used to cross-sectionally and retrospectively analyze secondary data from outpatient prescription claims for secondary care using Python 3.10**.**

**Participants:**

The current analysis transformed 21 776 350 outpatient prescription claims from health insured into 193 552 insureds.

**Results:**

Insureds using IQR were split into three classes: low, middle, and high risk. Based on the silhouette coefficient, the insureds of all classes were divided into three clusters. In all data for a period of four years, the first through third clusters, there were 21 799, 7170, and 19 419 insureds in the low-risk class. Middle-risk class had 48 348,23 321, 25 107 insureds, and 14 037, 28 504, 5847 insured in the high-risk class were included. For the first cluster of low-risk insureds: the total average cost of prescriptions paid by the insurance for the insureds was $211, the average age was 26 years, the average franchise was 88.5US$, the average number of medications and prescriptions were 409 and 62, the total average costs of prescriptions Outpatient was 302.5 US$, the total average number of medications for acute and chronic disease was 178 and 215, respectively. The majority of insureds were men, and those who were part of the householder's family.

**Conclusions:**

By segmenting insurance customers, insurers can set insurance premium rates, controlling the risk of loss, which improves their capacity to compete in the insurance market.

**Supplementary Information:**

The online version contains supplementary material available at 10.1186/s12889-023-15753-1.

## Strengths and limitations of this study

- ➢ Since the clustering is based on national health insurance data and the research sample is matched to the population, it can be applied to the entire country.
- ➢ The results of class-based clustering are expressed as average costs, allowing decision-makers to compare them to findings from other studies.
- ➢ Insureds are not covered for all drugs on their outpatient claims by their health insurance organization.
- ➢ The income information for each insured was approximate and relied on an artificial neural network's estimation, hence the results are less reliable.
- ➢ Assessing the risk without taking into account other factors and relying solely on the number of pharmaceuticals and prescriptions insured.

## Introduction

People in different countries pay for healthcare services through insurance, which refers to protection against the risk of racking up medical costs and other connected expenses [1]. It has been proposed that health insurance may increase access to health care of acceptable quality given the significant unmet need for high-quality healthcare among the general population and the considerable underutilization of healthcare facilities in many nations [2]. Meanwhile, the exorbitant cost of medical services, which is rising exponentially over time, is out of reach for low-income individuals in developing countries. Good insurance coverage can save people from financial hardship in these circumstances [3].

Also, as studies show in the short or long term, spending too much on health care can threaten the standard of living of a household [4]. In line with this subject, and according to the World Health Organization's report on the state of health spending, the amount spent globally on healthcare increased from US$ 7.6 trillion in 2016 to US$ 7.8 trillion in 2017, or approximately 10% of GDP and $1.080 per capita [5]. Additionally, the health industry is still growing faster than the overall economy. Real health spending increased by 3.9% annually between 2000 and 2017, above the economy's 3.0% annual growth. Between 2000 and 2017, health spending increased by 6.3 percent yearly for middle-income countries that are fast moving toward greater levels of spending, while the economy grew by 5.9 percent annually and these health costs in low-income countries soared by 7.8% each year [5]. In addition to this, over 5% of the public expenditures on the health sector during these years in 97 countries were made up of social and health insurance, which are crucial components of the health systems [5].

Furthermore, in more detail government budget allocations are the primary source of funding for around two-thirds of nations having social and health insurance. The percentage of social and health insurance in current health spending ranged from 1 to 2% in low income nations, 4.5% to 8.5% in lower-middle-income countries, and 16% to 20% in higher-middle-income countries [5]. As social and health insurance funding has increased, it is unclear what this means for the development of universal health coverage. Governments with social and health insurance systems do not appear to have superior population coverage with healthcare services when comparing GDP and government health spending per capita [5]. While patients are waiting for the payments to be made, the cost of health care, especially medicine, should not be such that it negatively affects their incomes or prevents them from providing other necessities in their lives [6]. Recent years have seen a significant increase in the importance of medication costs to policymakers around the world, both in terms of their absolute value and their proportion of overall health expenditures [7]. So, drug costs are typically the second or third component of health system costs in all countries after hospital admissions and physician visits [8]. The World Health Organization's report on the status of drugs in 2011 also states that total drug costs account for 1.41 to 1.63 percent of the gross domestic product depending on regions and income groups, with a significant difference of 0.2 to 3.8 percent of the gross domestic product between the lowest and highest income groups [9]. Even though $1.3 trillion is predicted to be spent globally on prescription medications in 2020. It is anticipated that these high spending rates will rise by 3–6% annually across the world [10].

The rising cost of pharmaceuticals has affected several stakeholders. Individual buyers are most directly susceptible, and many are forced to choose between paying for medicine or withholding treatment. Moreover, the cost of insuring people has increased for insurance firms. Government spending increases to provide funding for insurance coverage. Pharmaceutical firms, on the other hand, emphasize the necessity of generating adequate profit margins and resources for ongoing development and research [11]. The nation's economy and public health could suffer if pharmaceutical expenses are not properly addressed. In this regard, many developing countries lack adequate means to purchase medicine to meet poor demand. Additionally, factors such as drug price, household income, age, gender,social status and other related factors contribute to the problem [12]. Additionally, consumers with sub-optimal coverage tend not to save on services, which can result in a waste of insurance resources [13].

On the other side, health insurance as one of the providers of health services constantly going through fundamental changes as a result of issues including evolving rules and regulations, declining costs, developing technology, and establishing tariffs to respond to the changing conditions and requirements of the environment. Therefore, insurance companies are actively searching for new markets, luring in low-risk clients, and offering top-notch services. Health insurances are also particularly affected by business processes and technological changes. Due to the variety of consumers and their needs, this type of insurance, like other insurances, requires the storage and analysis of a significant amount of data. This type of data is gathered from a variety of sources, including health facilities, clinical settings, academic medical centers, electronic health records, and validated websites [14]. It is exceedingly challenging to adequately handle and evaluate all the data because it is stored in a variety of formats [15]. Grouping or clustering the vast health insurance data into a more manageable format is a crucial approach for resolving this analytical problem [16].

This is why health insurance providers have begun to divide up their clients into groups based on the traits that make them tick, employing data science advancements and placing an ever-increasing emphasis on going beyond the conventional and customary manner. Identifying these behavioral and demographic traits in customers and comprehending the patterns associated with them can help executive managers and policymakers better grasp the requirements of the customer’s demands. Among the data that are recorded and stored in the health insurance organizations, the data related to patients' claims is one type of data that is managed to keep on record and stored by insurance organizations. By looking at the findings of clustering methods, these organizations can uncover patterns and relationships between the data of insureds' outpatient prescriptions, their purchasing patterns, and the types of services they receive when grouped together with other insured individuals who share similar traits and assist in developing effective marketing plans for the health insurance sector.

Taking into account understanding, identifying, and categorizing the insureds based on demographic traits, risk type, type of health services, and costs can have a significant impact on the health markets with providers like health insurers, pharmaceutical companies, and the government. Because this inaccurate identification may lead to moral hazard by lowering the insured's willingness to contribute to the price of pharmaceutical services or increasing the insurer's tendency to make adverse selections [17, 18]. Both of these miss the mark of the fundamental goals of health systems, which are to promote health and protect people from healthcare expenses.

So, the main goal of study is to determine how well applying clustering analysis as a method of data mining to highly dimensional health insurance data (outpatient prescription claims) will allow us to find relevant clusters. We investigate cluster analysis in an effort to provide a response to this query. We find some intriguing trends that policymakers in insurance companies can employ.

## Methods

### Study design and setting

Secondary health insurance data were used to provide the essential statistical data for the current study, which is cross-sectional, retrospective in nature and focused on secondary care.

Collecting the required information was done by referring to the national's health insurance research center and through the insurance outpatient prescription claims of the insureds for the necessary analysis using Excel software 2016.

Due to the accurate recording of the demographic data and prescriptions from 2016, the research population consists of 21 776 350 outpatient prescriptions claims of the National Health Insurance Organization from 2016 to 2019. The population in this study is the same as the research sample, and the data from the sample will be clustered in that each outpatient prescription claim in this study was paid in 2016, 2017, 2018, and 2019. Additionally, the insured's income data was also derived using records from the Statistical Center of Iran on their overall income for 2019.

Following that, the medications that the health insurance company covered were identified. Three specialists divided medications into two drug groups for acute and chronic diseases based on the World Health Organization's definitions of acute and chronic diseases, which define chronic disease as having a protracted duration, typically progressing slowly, and not be transmitted from person to person. A condition that typically presents quickly and resolves in less than six months is referred to be an acute disease [19]. Due to the problem of overlapping medications specialists believe that when medicines are recommended for both acute and chronic diseases, they are placed in a group that is more frequently used and prescribed for that disease (acute or chronic).

### Clustering variables

The goal of cluster analysis is to identify significant data item divisions. By choosing features or sequences of groups that do so, a target function of interest can be optimized [20]. Because there is no prior knowledge of the variables in outpatient prescription claims clustering, it is necessary to extract and choose the most important characteristics from the initial data set [21]. As a result of the fact that the insured's registered data in the health insurance database is sparse, erratic, inaccessible, and noisy, it was attempted in this study employs every variable registered in the primary database in order to achieve the best clustering outcomes [22]. So, features of all data for all insureds in each cluster for a four-year period can be referred to demographic information, gender (male or female),and age (classified as [1–10], [10–20], [20–30], [30–40], [40–50], [50–60], and ≥ 60), main (being a householder or member of the family), the total average Insurance paid, the total average number of medicines for acute and chronic disease, the total average number of medicine, the total average number of prescriptions, the total average insurance paid and deductions, the total average franchise (co-insurance cost paid by insured), the total average deduction (deductions per prescription), the total average income (estimated income for each insured using an artificial neural network) are in order to adequately define each of the features listed in the clusters.

### Risk analysis of the insured

In view of the fact that the health insurance company does not keep track of any information relating to the insured's risk during the term of the insurance contract with the insured (such as records of hereditary diseases, high-risk behaviors, underlying conditions, income information, etc.) using data that is reliably documented in the health insurance database, an attempt has been made to determine the risk in this study.

In order to categorize the insured's risk assessment based on the number of medications and the number of prescriptions per insured, we divide the insureds into three classes—low, middle, and high—in order to evaluate specific sets of clusters. The insured's risks can be categorized in the following ways based on the theoretical foundation mentioned. Then we determine the interquartile range (IQR), which is the space between the 3rd and 1st quartiles of cluster sizes to determine the low, middle, and high risk thresholds. We acquired an IQR of 48 388–96 776 insureds. And we define low-risk clusters as being below the IQR, middle-risk clusters as being within the IQR, and high-risk clusters as being above the IQR. The entire subset of the cluster that K-Means produced is then subjected to analysis.

### Statistical analysis

Prior to applying the k-means method to cluster data, it should be noted that the initial data gathered from the insurance organization was complete and contained no missing data. The data collected also revealed that there were 21 776 350 outpatient prescription claims separated from one another every four years (2016–2019) based on the specific codes and 11 features of the outpatient prescription. Using these identification codes, the researcher converted the outpatient prescriptions to each insured. Simple mathematical operations like addition and multiplication were carried out with Python software by applying the required codes.

In accordance with studies, one of the most beneficial aspects of data mining is clustering, which helps identify groups, determine interest distributions and identify patterns in the data. A data set must be divided into groups (clusters) so that the points in each cluster are more similar to one another than the points in the other clusters. For example, dividing current insurers into specific categories and correlating a profile with each group can be important in future pricing schemes, for example, co-insurance rates [23].

Although several clustering algorithms have been created to evaluate data, it is still difficult to determine which approach produces the best and most appropriate amount of clusters with regard to various data sets [24]. Several healthcare data sets and distinct validation measures have been used by numerous researchers to test the clustering methods [25–28]. Only a few publications have evaluated the variability and performance of three different clustering methods using simulated data sets, and actual data sets as every data set is distinctive in some manner [29]. The fact is, many clustering algorithms have been created for the healthcare industry, but they were not assessed on various sets of crucial data. However, only experimental research of a particular healthcare data set was done to determine the relative importance of each approach. According to the literature on clustering, several clustering methods, including K-means, K-Medoids or Partitioning Around Medoids (PAM) have been developed for the analysis of healthcare data sets [30–32] and as no method for clustering is flawless and each method has advantages and limitations of its own, the best method for clustering can be employed depending on the goal of the study and the type of variables [33]. The k-means method clustering algorithms are renowned for their outstanding computational efficiency and very low temporal complexity when clustering large datasets are unlabeled [34, 35]. Also, when working with high-dimensional data, some features may be irrelevant while others may play a different role in clustering. As a result, different variables within a cluster may have contributed differently to the cluster structure. The significance of each variable to each cluster must be considered for improved clustering. The best option might be the K-means algorithm with configurable weighting [36].

### K-means algorithm

The objective of the k-means clustering approach is to identify the natural partition of the data set into k clusters and noise. Take into consideration a data set with N data that is n-dimensional containing x. With the indicator vector μ*i* having the value,…,1 k, we know that there are k discontinuous clusters with *N j* data points. The clustering function from Eq. (1)'s clustering function sum of squares is what the k-means algorithm seeks to reduce:

1 $$J={\sum }_{j=1}^{k}{\sum }_{i=1}^{x}{\Vert {x}_{j}^{i}-{c}_{j}\Vert }^{2}$$

1. That || || the measure of the distance between the points and ${c}_{j}$ is the center of the j cluster.
2. Random selection of C point for initialization of cluster centers.
3. Determine a criterion for calculating the distance between data that we use Euclidean distance to get the closest cluster center for each sample. All samples are in C clusters at the conclusion of this stage.

Choosing a criterion for measuring the distance between data is one of the key difficulties in clustering. The most popular and extensively used method for determining the separation between two objects is the Euclidean distance. Discrete cluster analysis based on Euclidean distance is carried out in clustering methods. These Euclidean distances are computed from one or more core variables that the algorithm generates and modifies. It is possible to determine the clustering criterion that is used to measure the distance between observations and kernels. The clustering of observations is done so that each observation can only belong to one cluster. Clustering studies do not have any dependent variables and we do not profile a specific characteristic like classification studies [37].

Initial centroid values and the number of clusters will be defined using the k-means technique. Using the shortest distance between a centroid and a data point, the algorithm then divides the input dataset into k clusters. After each iteration, the algorithm dynamically adjusts the centroid values. This cycle repeats until the centroid value stays constant [38].

That Eq. (2) yields the Euclidean distance between two points in n-dimensional space, x and y.

2 $$d\left(x.y\right)=\sqrt{{\sum }_{i=1}^{n}({x}_{i}}-{y}_{i})$$

4. We determine the new center of gravity and update its value for every cluster.
5. In order to complete the algorithm, repeat steps 2 and 3(15).

As previously stated, the objective of this algorithm is to maximize distance between components of different clusters while minimizing the distance between components of a cluster. After cleaning data prior to clustering, the features must be normalized to prevent variables with bigger units from dominating the distance features. By looking at the most prevalent features inside specific sets of clusters and performing a high-level analysis across all clusters, we can evaluate the effectiveness of our insured clustering. We can test our clusters' ability to capture significant situations at a higher precision by looking at each individual cluster. We may analyze the broad patterns and conditions our clusters capture at a higher level by looking at all of the clusters at once [39].

### Clustering validation

The main objective of the assessment indicator is to assess the reliability of the algorithm. While creating a clustering algorithm, assessment indicators can be categorized into two categories based on the test data: internal evaluation indicators and external evaluation indicators. The algorithm's validity is examined using internal data from the internal evaluation. Yet, when two algorithms' ratings differ based on internal evaluation indications, it is impossible to determine which algorithm is superior. The criterion for testing methodologies, known as the external evaluation, uses external data to assess the algorithm's viability. In the absence of any additional external data, the Silhouette and David Bouldin criteria were applied for the study's external evaluation.

During internal validation, just the data included in clustering are used. Calculating an index that is intended to gauge how effectively the clustering corresponds to the data is the typical internal validation procedure. These indices frequently take advantage of the data's proximity structure, for instance, by determining the homogeneity and/or dispersion of the clusters. The silhouette coefficient is an illustration [40]. Similarly, for high-level analysis of all clusters we evaluate the appropriateness of the number of clusters we choose using silhouette analysis. The silhouette score measures how well samples are clustered with other samples that are similar to them in order to assess the quality of clusters produced by clustering algorithms like K-Means [30]. As each data point's silhouette score is generated, the following distances for every observation a part of every cluster must be determined:

The observation's average distance from every other data point in the cluster. A mean intra-cluster distance is another name for this distance. with a, the mean distance is indicated.

If we know that insured i belongs to cluster Ci and that its silhouette score is s(i), we may calculate this. Let a(i) be the average intra-cluster distance for insured i and b(i) be the average inter-cluster distance between the insured i and all points in the cluster that is closest to cluster Ci. Then, let *d* (*i*, *j*) be the Euclidean distance between some insured j's eigenvector portrayal and insured i's eigenvector illustration. thus, the silhouette score can be demonstrated using the s(i) calculation above that a silhouette score falls between -1 and 1.

$${a}_{i}=\frac{1}{\left|{C}_{i}\right|-1}{\sum }_{j\epsilon {C}_{i.i\ne j}}d(i.j)$$

$${b}_{i}={min}_{k\ne i}\frac{1}{\left|{C}_{k}\right|}{\sum }_{j\epsilon {C}_{k}}d(i.j)$$

$$s\left(i\right)=\left\{\begin{array}{c}\frac{b\left(i\right)-a(i)}{\mathrm{max}(a\left(i\right).b\left(i\right))}. if \left|{c}_{i}\right|>1\\ 0. if \left|{c}_{i}\right|=1\end{array}\right.$$

Higher values indicate definite or distinct cluster assignments, whereas lower silhouette scores indicate confusing or potentially unsuitable cluster assignments.

And for the David Bouldin index, the low value of this index indicates that intra-distance is minimal and inter-distance is quite big, resulting in an optimal clustering. That this index's value is determined using the following formula for each class.

$$DB=\frac{1}{K}\sum_{i=1}^{k}{max}_{i\ne j}(\frac{{\sigma }_{i}+{\sigma }_{j}}{d({c}_{i}.{c}_{j})})$$

K is the number of clusters, ${\sigma }_{X}$, the average distance between any data in cluster x and ${C}_{X}$, ${C}_{X}$, is the center of cluster x, $d({c}_{i}.{c}_{j})$ denotes the distance between ${c}_{i}$ and ${c}_{j}$

Then, as a kind of feature scaling, min–max scaling is used for normalization. The Min–Max scaling rule is demonstrated in Eq. (1). All features were converted to binary with '0' and '1' values [41].

$$Min-Max\;scalingX=\frac{X-\min(x)}{\max\left(x\right)-\min(x)}$$

In Eq. (1), max(x) and min(x), respectively, represent the maximum and minimum values of X. To create a data set with "1" as the overall standard deviation, data are adjusted in the 0–1 range and standardized. The mean value is additionally normalized to "0" using the centering approach. The identical size for all features is made possible by scaling and centering, which speeds up learning for clustering and prevents over-fitting [42]. Next, individuals are clustered in Python 3.10 using 11 features chosen from the data of insured claims.

We also utilize the grid search approach to build the ideal parameters from the default parameters of each cluster, which allows us to compare the cluster results based on the best parameter and illustrates the effects of hyperparameters for future research and better decision-making [43]. The examined insurance claim data undergo a number of preprocessing stages, during which features are chosen. After choosing the pertinent features, it is given as input to the machine learning clusters. In order to improve the cluster's effectiveness, the grid search does parameter tuning. After optimization using the aforementioned technique, the silhouette score for the clusters will be calculated for each insured using the k-means method.

Since there was no household income of the insured like other variables in the health insurance data to calculate it, the insured were identified and categorized in accordance with the responses to the codes connected to the payment of insurance premiums in order to determine the household income based on the cost-income questionnaire of the statistical Centre of Iran. After finding the relevant codes, the insureds under other insurances were excluded from the samples. After all the procedures were finished, a sample of 38 319 individuals was studied to estimate the income of the insured covered by the Iran Health Insurance Organization using an ANN technique. Data fitting and attempting to obtain the best fit by adjusting the network's parameters are two uses of artificial neural networks. In general, it may be argued that neural networks are composed of layers of neurons, which are connected to the outside world by their inputs and produce the external world through their outputs [44].

The neural network's first step focuses on identifying and analyzing the factors that influence income. Since the model's ultimate objective is to determine the income of insured people using already-existing indicators like age and sex as well as data about the income of 38 320 individuals who had health insurance that was taken from the household income-cost questionnaire, the output index, thus, a figure of 19 3552 is produced for the expected annual income. Once these procedures have been completed, the neural network has been trained using various permutations depending on the algorithm for learning the number of layers and neurons. The transfer functions for the hidden layer and the output layer, which are linear and hyperbolic tangent functions, respectively, in all of these executions of the network's Python software implementations, have not changed. There are 50 neurons in the first layer, 50 neurons in the second layer, batch normalization in the third layer, 50 neurons in the fourth layer, 20 neurons in the fifth layer, and 1 neuron in the final layer in the hidden layer, and each layer has undergone Relu activation.

Fifty-five percent of age and 45% of gender were significant factors when using Shap's neural network's sensitivity analysis technique. Additionally, the MSE for the training data was 8.9 *10 ^12^, for the test data 2.9* 10 ^16^ and the learning rate was 10 ^−4^.The high values of these numbers point to the shortcomings of the indicators used for the accurate assessment of income. This means that based on age and sex alone, income cannot be accurately predicted.

Since the amount of data is based on the Iranian common currency, the cost and income variables are all adjusted according to the average exchange rate stated by the central bank for the four years 2016–2019 after executing all the processes for clustering and estimating incomes [45]. (1 dollar = 12,448.14 Rial).

### Patient and public involvement

The design, conduct, reporting, or dissemination strategies for this study did not involve patients or/and the public.

## Results

Following data cleaning and the removal of inaccurate, incorrect, and irrelevant data due to user input errors, storage or transmission problems, or different data dictionary definitions from stores with similar objects, the resulting data from outpatient prescription claims were 21 776 350 in 4 consecutive years, which were then converted into 193 552 individuals for clustering. Then based on IQR of 48 388–96 776 insureds, we established low, middle, and high-risk classes according. The number of optimal clusters of insureds within each class was defined by the silhouette coefficient's value.

Tables 1, 2 and 3. The first cluster of low, middle, and high-risk classes is revealed by descriptive statistics for a 4-year period. Based on the 11 features taken into consideration, the findings are the total average for all insureds in the clusters. The total average cost paid by the insurance (the amount from which franchise and deduction are subtracted) for the 21 799 insureds in the first cluster of low risks class is $211, with a standard deviation of 72.16; the minimum and maximum payments paid by the insurance for the insureds are $0.38 and $320, respectively. For 25% of the insureds, the insurance only paid less than 162 dollars. 50% of the insureds were covered by the insurer for $220. Additionally, the insurance only paid less than $271 for 75% of the insureds. The total average age is 21 years (Std = 19.41), The total average age of all insureds is 5 years minimum and 101 years maximum. 25% of the insureds are under the age of 14, 50% are within the age of 20, and 75% are below the age of 33. The total average number of medications for the insureds is 409; the minimum and maximum numbers are one and 144,108, respectively. 25% of the insureds have less than 174, 50% have 318, and 75% have fewer than 507 medications. The insureds have a total of 62 prescriptions on average; the lowest and greatest numbers are 1 and 171, correspondingly. Of the insureds, 25% have fewer than 46, 50% have 62, and 75% have less than 79 prescriptions. The total average cost of a prescription for insureds (sum of insurance paid & deductions plus franchise sum) is $303; the lowest cost is $0.38 and the highest is $1979. 25% of insureds had prescription costs below $230, 50% $314, and 75% were under $389. The total average franchise (the amount paid by insureds) is $88.58 (Std = 35.10). The insured's minimum and maximum franchise amounts are 0 and $1316. 75% of the insured paid less than $114, 50% paid $91, and 25% paid less than $65.5.

**Table 1:** K-means method descriptive statistics for the first cluster of the low risks class

Features	Number of insured	Mean	Std	Min	%25	%50	%75	Max
^a^Insurance paid sum	21,799	211.46	72.16	0.38	162.03	220.11	271.30	320.85
age	21,799	26.73	19.41	5	14	20	33	101
Medicine sum	21,799	409.24	1301.79	1	174	318	507	144,108
Prescription sum	21,799	62.75	24.43	1	46	62	79	171
^a^Sum of Insurance paid & Deductions	21,799	214.69	73.55	0.382	164.51	222.88	274.95	663.06
^a^Deductions _sum	21,799	3.23	9.49	-29.69	0	0	1.68	386.32
^a^Franchise sum	21,799	88.58	35.10	0	65.50	91.20	114.27	1316.85
Medicine for Acute disease	21,799	178.31	122.03	0	85	158	246	2247
Medicine for Chronic disease	21,799	215.19	1289.71	0	32	102	247	143,919
^a^Income	21,799	4342.68	2460.09	1950.51	1950.51	3644.58	6494.81	10,175.47

^a^ Every cost is expressed in US dollars (USD)

**Table 2:** K-means method descriptive statistics for the first cluster of the middle risks class

Features	Number of insured	Mean	Std	Min	%25	%50	%75	Max
^a^Insurance paid sum	48,348	597.87	186.888	320.87	436.62	570.52	744.58	991.01
age	48,348	37.42	21.46	5	20	36	49	101
Medicine sum	48,348	1329.30	25,841.61	1	534	906	1517	5,619,674
Prescription sum	48,348	143.0812	50.55	1	106	136	174	446
^a^Sum of Insurance paid & Deductions	48,348	604.77	189.55	318.29	441.37	577.39	752.81	1372.24
^a^Deductions _sum	48,348	6.90	18.53	-127.24	0	0.61	5.90	521.02
^a^Franchise sum	48,348	248.34	85.09	0	180.13	236.40	308.81	1699.02
Medicine for Acute disease	48,348	343.93	262.18	0	182	302	451	27,204
Medicine for Chronic disease	48,348	824.40	3830.43	0	210	497	1040	805,230
^a^Income	48,348	5909.84	2589.50	1950.51	3644.58	6645.14	7780.66	10,175.4

^a^ Every cost is expressed in US dollars (USD)

**Table 3:** K-means method descriptive statistics for the first cluster of the high risks class

Features	Number of insured	Mean	Std	Min	%25	%50	%75	Max
^a^Insurance paid sum	14,037	2422.37	4713.89	991.0765	1185.85	1473.77	2091	169,879.3
age	14,037	55.68	8.07	13	51	55	59	96
Medicine sum	14,037	5464.88	155,457	100	1892	3174	4980	17,740,701
Prescription sum	14,037	297.78	130.18	31	213	274	352	1736
^a^Sum of Insurance paid & Deductions	14,037	2444.30	4730.27	952.70	1197.35	1490.58	2114.92	169,880.8
^a^Deductions _sum	14,037	21.92	113.06	-200.41	0.12	2.52	13.66	6804.11
^a^Franchise sum	14,037	774.57	1062.21	10.49	477.06	592.91	809.34	59,589.09
Medicine for Acute disease	14,037	736.76	586.54	0	355	589	954	8953
Medicine for Chronic disease	14,037	4288.92	150,080.3	12	1204	2360	4047	17,740,548
^a^Income	14,037	5122.63	746.18	1290.11	4696.32	5046.10	5575.48	10,130.87

^a^ Every cost is expressed in US dollars (USD)

One hundred seventy-eight and 215 medications, respectively, are used on total average to treat acute and chronic illnesses. For acute illness, the minimum and maximum number of medications was 0, 2247, while for chronic illness, it was 0,143919. For acute diseases, 25% of insureds had less than 85, 50% had 158, and 75% had less than 246 medications. This quantity was smaller than 32, 247, and equal102 to the respective percentages of 25%, 75%, and 50% of those insureds for the medication of chronic disease. And the same applies to the interpretation of the results for the second and third clusters in the low risks class, as well as the first through third clusters in the middle and high risks classes. (see Appendix).

Table 4. The silhouette coefficient can be used to verify the K-means clustering. In Table 2, the various Silhouette coefficients for the clusters are shown. The Silhouette Coefficient provides information on the effectiveness of the K-means algorithm's clustering. A high positive number (+ 1) denotes a strong clustering of the data and a negative number (-1) and values around it indicate poor clustering. The Silhouette Coefficient value tells us how efficiently the K-means algorithm clustered the data for each k (k = 1, 2, 3). We can observe that the Silhouette Coefficient for each cluster indicates that the clustering was successful. Truth be told, a range of 2 to 20 clusters was taken into consideration in order to get an efficient result of the silhouette coefficient near one. As the number of clusters rises, the silhouette coefficient decreases until it eventually reaches zero.

**Table 4:** Silhouette coefficient for each class and its associated clusters

Class	Clusters	Number of insured	Silhouette Coefficient
Low risk	Cluster 1	21,799	0.70
	Cluster 2	7170	
	Cluster 3	19,419	
Middle risk	Cluster 1	48,348	0.68
	Cluster 2	23,321	
	Cluster 3	25,107	
High risk	Cluster 1	14,037	0.71
	Cluster 2	28,504	
	Cluster 3	5847	

Table 5. As a result of our experiment, K = 3 in each class had the Davies Bouldin value. When the intra-distance (the distance among objects within a particular cluster) and the inter-distance (the distance across clusters) are both small, this is known as a small Davies Bouldin value, which can point to a successful clustering.

**Table 5:** Davies–Bouldin indicator for each class

	Class		
Evaluation indicator	Low risk	Middle risk	High risk
Davies–Bouldin indicator	5.80	4.51	3.78

**Fig. 1:**
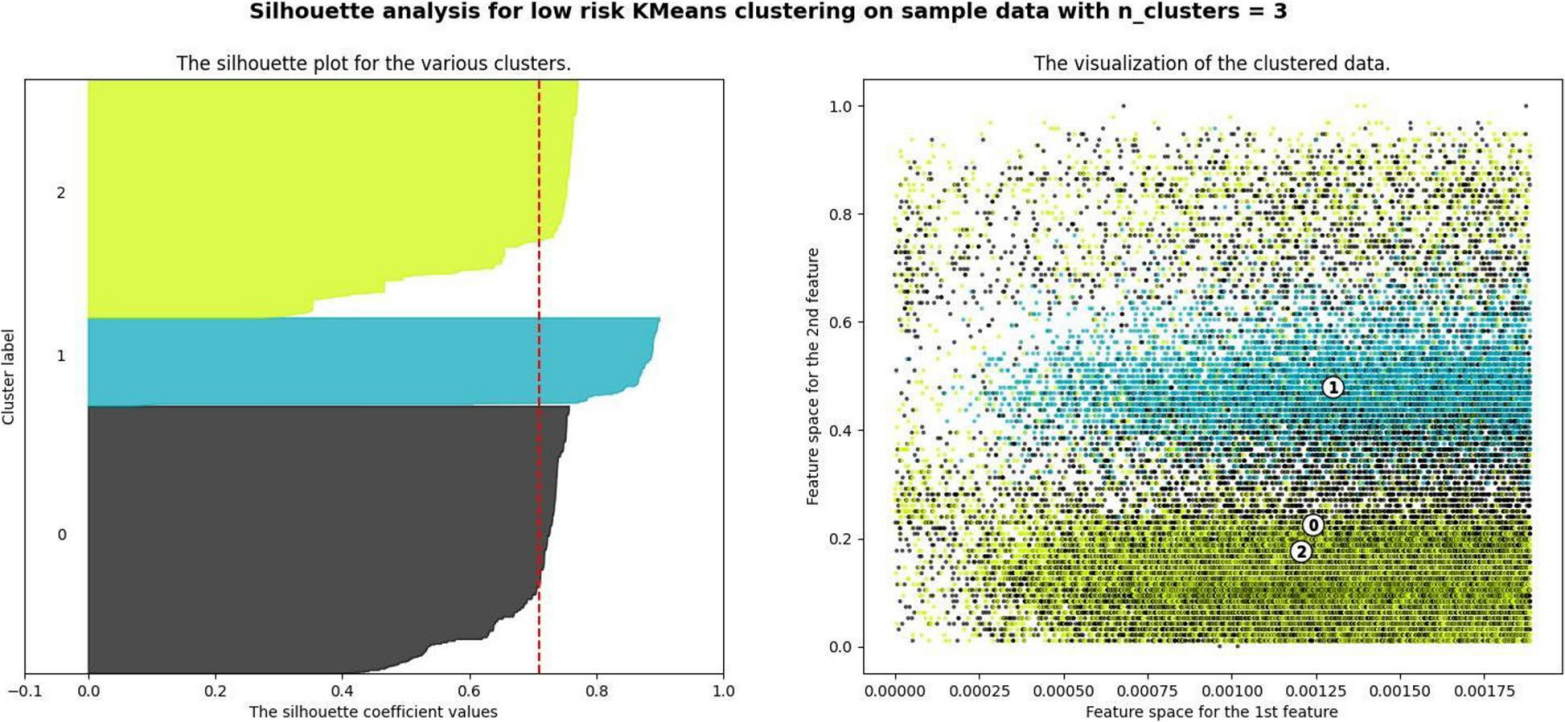
Silhouette plot produced empirically for low risk k-means clustering

**Fig. 2:**
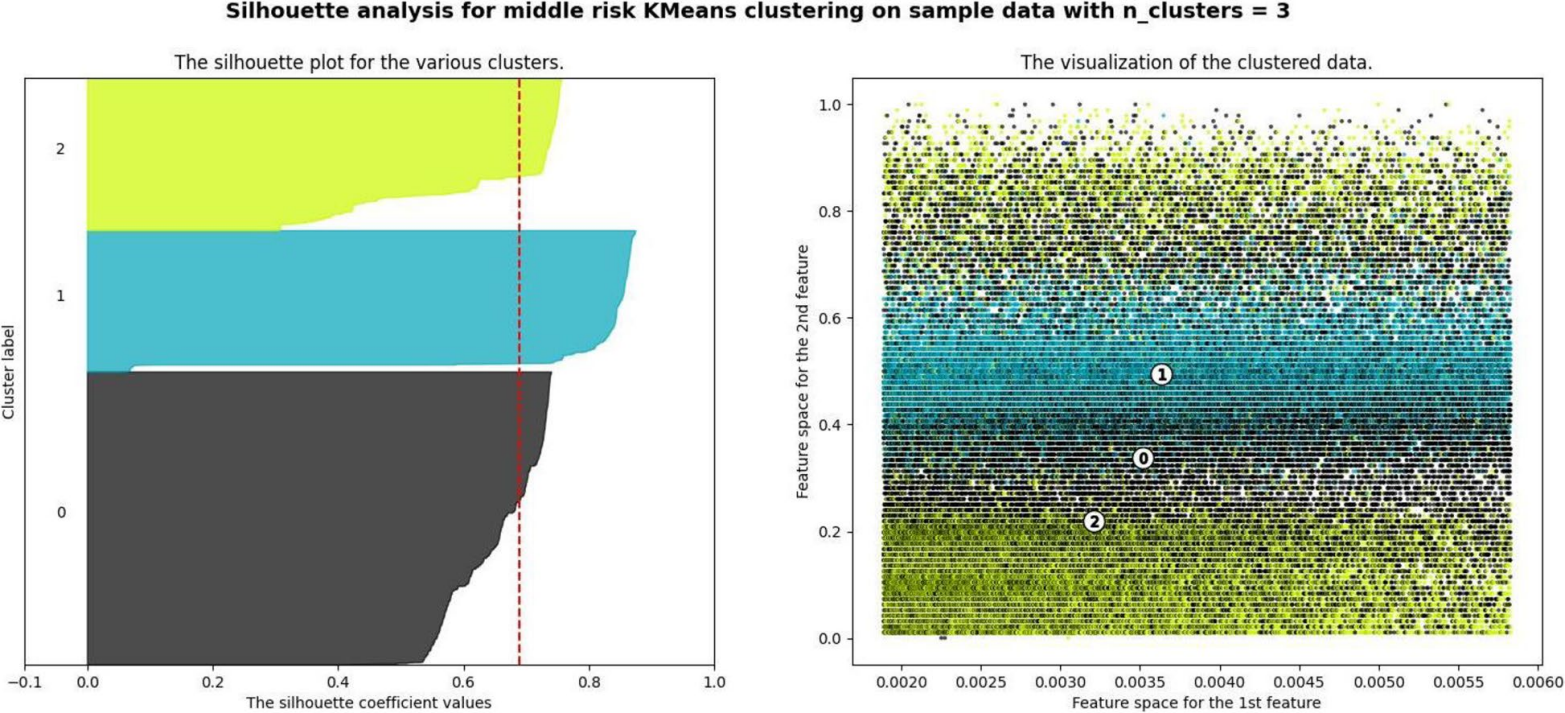
Silhouette plot produced empirically for middle risk k-means clustering

**Fig. 3:**
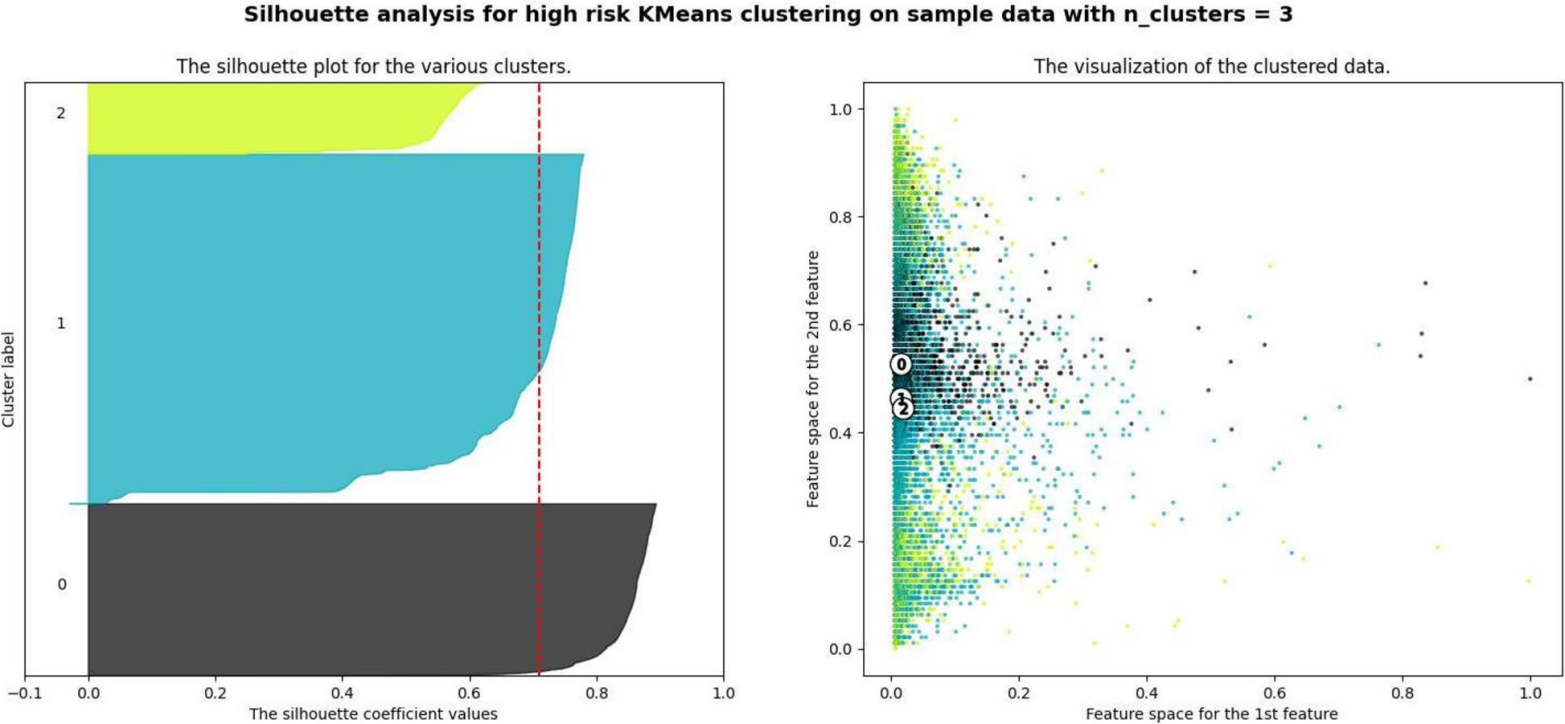
Silhouette plot produced empirically for high risk k-means clustering

Figures 1, 2 and 3 Illustrate silhouette plots for each class and its clusters. The cluster silhouettes are displayed in random order on the silhouette plot. In addition, it adds white space and has the ability to change the colors of subsequent clusters. A silhouette plot is shown on the left. The numbers for the silhouettes are shown along the x-axis, and the height of each silhouette represents the number of points in each cluster. The color-coded data points in the right subplot are represented by the clusters' hues.

The plots related to each class and the number of their clusters are drawn in a two-dimensional space of the insured's age, insurance paid sum, and the number of insureds in each cluster because it is impossible to draw clustering plots in an 11-dimensional space of features. A large percentage of insured data points must have a positive silhouette coefficient (+ 1) for a group of clusters' silhouette plots to be considered properly formed, and each cluster's silhouette plot must be roughly smooth in shape with few spikes, as these spikes could represent vague or possibly incorrectly designated data points. In fact, by examining the silhouette plots in Figs. 1, 2 and 3, we see that our method has well-formed silhouette plots with the exception of the first cluster of the low-risk class despite the silhouette coefficient being close to one. In addition, due to the different values of the cluster and the high silhouette coefficient, the clusters' thickness in the plots is not uniform. As well as the presence of clusters below the mean silhouette score (The red-dotted line displays the average score) indicates a suboptimal n cluster. Cluster 1 in the high-risk class displays value below the average score for the provided data. However, the silhouette coefficient for the first cluster in the high-risk class, is near 1.

**Fig. 4:**
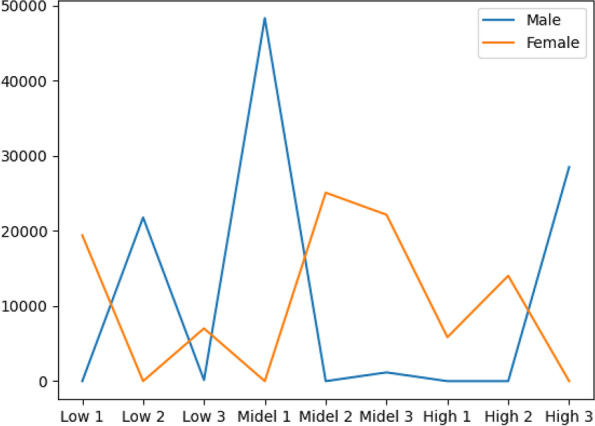
The number of insureds in each class, broken down by gender

Figure 4 It displays the gender-based distribution of insureds in each cluster for each class. Men in the first and second middle- risk class clusters took the most and the least amount, respectively, as seen by the graph. And for women, in contrast, the second cluster of middle-risk class has the largest number and the first cluster has the lowest number.

**Fig. 5:**
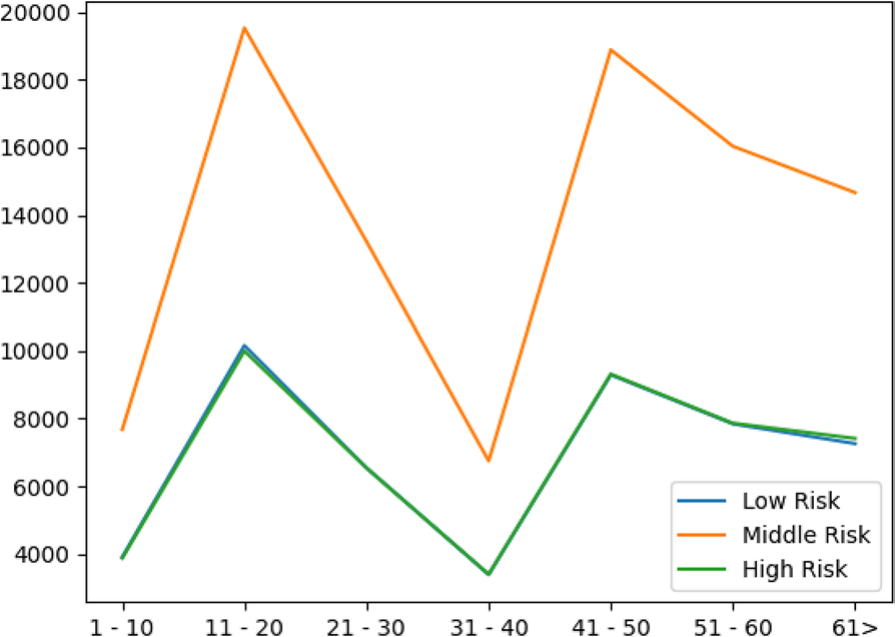
The number of insureds in each class, broken down by age

Figure 5 The number of insureds of each age category in each class. The graph shows that for all three classes, the age groups of 11–20 and 41–50 have the greatest numbers of insured people, while the age group of 31–40 has the smallest number

## Discussion

The collection of a significant volume of heterogeneous data from numerous sources—which is typically available in an unstructured format—increases the processing time required to process the data and analyze it. Finding useful information from unstructured raw data is the goal of the process known as data mining. Its goal is to use data to draw conclusions and identify trends that can be applied to the development of successful business plans for a variety of roles. This will assist businesses in reaching their goals and improving decision-making. Data mining can be used to automatically forecast outputs in accordance with patterns, create groups based on discoveries, make predictions that are probably based on findings, and visually represent heretofore unrecognized groups of data.

Unsupervised learning as one of the data mining approaches involves a set of data without an associated assortment of labeled outputs. A collection of unlabeled outputs must be analyzed by the unsupervised learning algorithm in order to find patterns that are concealed within. The main goal is to make each cluster different from the others by reducing proximity between data points that belong to similar groups. The fact that clustering methods in an unsupervised learning approach dynamically divide the dataset into groups according to its common features and aid in the discovery of characteristics that may be potentially helpful for categorization are two of its main benefits [46].

Clustering analysis is utilized to determine groups to achieve the goal of later allocating additional items (objects) to these categories [47, 48]. Accordingly, the current study looked at 21 776 350 outpatient prescription claims made by health insureds and converted them into 193 552 individuals. Then based on IQR of 48 388–96 776 insureds were split into low, middle and high-risk classes using the k-means method into three clusters relying on silhouette coefficient with 11 features considered. First through third clusters of data for a four-year period showed 21 799, 7170, and 19 419 insureds in the low-risk class. 48 348, 23 321 and 25 107 insureds were in the middle-risk class, whereas 14 037, 28 504 and 5847 covered insureds were in the high-risk class. K-means clustering was improved for Fashoto et al. in order to address issues with the National Health Insurance Scheme, this study developed an enhanced real-time assignment K-means clustering method that may be applied to classify health insurance claims [49]. When it comes to clustering based on classes, the customer risk analysis by Bi et al. is divided into 4-level and 5-level risk degree analyses [50]. Furthermore, Zahi et al. using K-means, formed 3 clusters include the active spouse population, retirees or their spouses, and the child cluster. They used the silhouette coefficient as a validation metric in addition to empirical validation to make sure that the clusters formed served the study's intended purpose [51].

Regarding the statistical findings from the clustering of the insureds, in the first cluster of low-risk insureds, the total average for each feature the total average cost of prescriptions paid by the insurance for the insureds was 211$, while this amount is $597 for the first cluster of middle-risk class and $2422 for high-risk class. This may be in line with the assumption that an individual's insurance will cost more to cover the higher risk. The majority of insureds were men and those who were part of the householder's family for the first cluster of low and middle risk and were women and householders in the first cluster of high-risk class. The average age was 26 years for insureds of the low-risk class, that is 37 years old for insureds in the middle-risk class and 55 years old for high-risk class. This indicates that high risks are older than established than middle and low-risks, on the basis of insurance economy concepts. This issue was confirmed by Kelly et al., who demonstrated in their study that age is a significant factor in insurance pricing and risk classification. As an individual becomes older, they incur more costs for the insurance provider and pay higher premiums [52]. As well as the total average number of medicines and prescriptions was 409 and 62 for low-risk class, 1329 and 143 for middle-risk class, 5464 and 297 for high-risk class. The number of prescriptions and medications rises in proportion to the insured risk. Besides that, the total average franchise for all the insureds in the low-risk class was 88.5US$, for middle-risk class 248 US$ and for high-risk class 774 US$. To put it another way, the insured pays more for the outpatient prescription as the risk of him\her increases, and the insurer contributes less.

Consequently, the average total costs of prescriptions Outpatient (sum of insurance paid and deductions plus franchise sum) was 302.5 US$ in the low risk class, 853.11 US$ in the middle risk class and 5504.84 US$ for the high risk class. Calculating the average cost for one insured after dividing the overall average cost by the number of insureds in each cluster and class yields the following results; for an insured in the first cluster of high-risks, the cost of an outpatient prescription is 22 times more than it is for an insured in the first cluster of middle-risks, and it is 27 times higher than it is for an insured in the first cluster of low-risks. In Liao et al. study the K-means approach was used to group 18 380 identified patients into 4 clusters, and the average cost variations between the 12 months before and after hemodialysis periods revealed an increase in costs for the first and fourth clusters and a drop in costs for the second and third clusters [53].

It is also possible to determine the prevalence of acute and chronic diseases among the individuals in each cluster by selecting the kind of medications for acute and chronic diseases as one feature among other variables of the insured outpatient drug prescriptions. As it pertains to chronic and acute disease medicines, the total average number of medications for acute and chronic disease was 178 and 215 respectively for low-risk class. 343 and 824 for the middle-risk class, 736 and 4288 for high-risk class. It may be inferred from the number of acute and chronic disease medications that there were more medications for chronic diseases in all three groups of insureds. This finding is important for policymakers because prescribing and paying for medications that maximize the welfare of the insured (the amount of out of pockets) and limit the financial expenses of insurance can be accomplished by using clustering in terms of pharmaceuticals for acute or chronic disease (reducing costs) [54].

According to the results of the current research and the studies stated, it can be discussed that clustering techniques are widely employed in any field, especially health insurance. The ideal k number of clusters can be determined using the insured segmentation models. However, it is not always possible to transfer the ideal model output into commercial outcomes. To get the most out of the model output, you need to know when to make concessions. It can be sensible to choose to have three insured segments (class), regardless of the magnitude of the insured portfolio, but it's crucial to remember that, despite the similarities among insureds in a cluster, insured uniqueness should still dominate. As well maximizing insured value is essential to thriving in today though insurance business environment. With increased insured expectations, insurance businesses must be able to distinguish between the most profitable and least profitable customers. By doing this, insurance businesses are guaranteed to remain relevant and match insured requests while encouraging growth. Further insurers are able to deliver an accurate calculation of insurance premiums, actuaries and more guided customer behavior and needs by having an understanding of when consumers join the insurance company and how they move throughout their customer lifecycle.

As a final point, there is relatively little research on this subject that can't speak with certainty about the impact clustering has on healthcare expenditures as well as the impact it has on enhancing the quality and equity of access to healthcare. For instance, Zhang et al. employed the Lorenz curve and the Gini coefficient to cluster patients in their study on the expenses of rural health care in China, and they came to the conclusion that policymakers should direct these patients to adequate health services and quality management in order to cut down on these patients' needless medical usage [55]. The characteristics of outpatient drug prescriptions, including the consumption of medicines for acute and chronic diseases, demographics such as age, gender and the total average income, the total average number of medicine, the total average number of prescriptions, the total average insurance paid and deductions, the total average franchise (co-insurance cost paid by insured), the total average deduction (deductions per prescription), and type of risk, can be said to help policymakers and insurers accurately meet the needs of the insureds and patients because the clustering method identifies patterns in the data. Although the literature on health economics indicates, evaluating the insured's risk increases the likelihood of cream skimming by insurance companies, one can stop from happening adverse selection resulting from the asymmetry of information and the continuation of high-risk individuals in health insurance and the exclusion of low-risk individuals.

Furthermore, the integrated consideration of these features in similar insured groups by identifying the patterns of such individuals can reduce the moral hazard brought on by excessive service consumption by insureds who reduce their health-related behaviors and do not contribute to their costs [56] that is avoidable through varying deductibles or co-insurance, raising the amount of preventative care that high-risk insured groups are covered for [57]. Besides that, in the case of "cream skimming" in private and public health insurance organizations, it is possible to stop inequality in access to and financing of healthcare services by introducing premium rate limitations that require insurers to charge the same premium for all individuals within specific risk classifications, risk adjustment premium subsidies and risk sharing, and ultimately enhancing the main aim of healthcare systems, “health promotion”[58].

### Suggestions and future research

We can get the conclusion that clustering is a challenging process with numerous steps requiring important considerations. These decisions all have an effect on the final clusters that are produced. The clustering studies, regrettably, do not offer clear guidelines; instead, much depends on the researcher's expertise and judgment.

Whereas every data mining technique has its advantages and disadvantages and heavily depends on the type and quality of the data, this study has attempted to accomplish everything that is necessary for accurate and improved clustering of the insureds. The application of clustering approaches in health insurance will give decision-makers possibilities and solutions to monitor insurance coverage overall and health insurance specifically, gain a greater understanding of insured activities, and forecast the behavior of new ones. Additionally, by implementing various customer strategies, insurance organizations may better identify various customer groups, and market sectors and develop strategies to expand the insurance industry.

On the basis of the clusters that have formed, a future study in this area is possible. In upcoming research, K-means can be contrasted with its vast variety. The developed clusters can also be utilized to examine co-payments and reimbursement modeling within each cluster in order to further investigate healthcare-related concerns. The same is feasible to carry out a study by comparing the outcomes of traditional and modern clustering techniques in health insurance.

## Conclusion

The segmentation and separation of consumers based on their behavior and needs are currently the most crucial action of the health insurance organization as the steward of health care services. This action is taken to increase the quality and quantity of health insurance, ensure full and equitable coverage of healthcare services, and lower the share of health costs borne by the general public. In addition to these concerns, questions can be what number of distinct insured types does a health insurance organization have? The thought of this question often occurs to insurers. Its underlying idea is assured clustering. The purpose of insured clustering is to classify insureds into groups that have similar features. By clustering the health insurance clients into similar groups, the insurance company can decide to charge each of those groups a different price instead of charging everyone the same rate, helping boost profits as those who would use the insurance more (prone to health problems) can be charged more. The best results, such as effectively targeting clients and enhancing the customer experience, etc., might be achieved by insurance firms with the use of effective clustering by developing unique and appropriate tactics for each group of customers correspondingly.

Depending on the activities, the health insurance firms can then look more closely at their original subset of clients owing to segmentation. In addition, regardless of how complicated the model is, it is always important to take into account the internal knowledge of the customer and of the sector. This is crucial in the health sector, especially if the results are to be used in the system in such cases. Given the assumption that in a realistic situation involving health concerns, a client who the model assumes falls into a specific group based on certain criteria, does not correspond to the profile. It is crucial to always take a meticulous and considerate approach from findings to action in this field of work because it involves the health of the insureds. Focusing on a customer's health state does not assure the kind of response that will be received; instead, preventive or cautious activities geared should be used as the strategy. Using customer segmentation to its fullest potential can also help health insurance providers maintain a committed customer base by enabling insureds to not only develop a relationship with the health insurance business.

## Supplementary Information

**Additional file 1: Appendix table S1.** K-means method descriptive statistics for the second cluster of the low risk class. **Appendix table S2.** K-means method descriptive statistics for the third cluster of the low risk class. **Appendix table S3.** K-means method descriptive statistics for the second cluster of the middle risk class. **Appendix table S4.** K-means method descriptive statistics for the third cluster of the middle risk class. **Appendix table S5.** K-means method descriptive statistics for the second cluster of the high risk class. **Appendix table S6.** K-means method descriptive statistics for the third cluster of the high risk class.

## Data Availability

Data are available upon reasonable request. shekoufehmomahhed@gmail.com will be responsible for any information about the data.
