# Supplementary material for: K-means clustering of outpatient prescription claims for health insureds in Iran
Source: BMC Public Health. 2023 Apr 28;23:788. doi: 10.1186/s12889-023-15753-1 (PMC10142779; doi:10.1186/s12889-023-15753-1)
Supplement: Supplementary file 1 — Additional file 1: Appendix table S1. K-means method descriptive statistics for the second cluster of the low risk class. Appendix table S2. K-means method descriptive statistics for the third cluster of the low risk class. Appendix table S3. K-means method descriptive statistics for the second cluster of the middle risk class. Appendix table S4. K-means method descriptive statistics for the third cluster of the middle risk class. Appendix table S5. K-means method descriptive statistics for the second cluster of the high risk class. Appendix table S6. K-means method descriptive statistics for the third cluster of the high risk class. [file 12889_2023_15753_MOESM1_ESM.pdf]

Appendix

K-means method descriptive statistics for the second through third cluster of the low, middle and high risk classes. An example of the interpretation of each table is given in the text for the first cluster of the low risk class.

Appendix table S1 K-means method descriptive statistics for the second cluster of the low risk class

Features	Number of insured	Mean	Std	Min	%25	%50	%75	Max
*Insurance paid sum	7170	221.50	67.18	6.63	173.54	230.69	277.87	320.86
age	7170	51.17	7.19	28	47	50	55	97
Medicine sum	7170	517.97	364.29	1	284	418	638	4425
Prescription sum	7170	64.27	23.36	1	47	63	80	178
*Sum of Insurance paid & Deductions	7170	224.96	68.78	6.63	176.01	234.54	281.24	706.57
*Deductions _sum	7170	3.46	11.20	-17.55	0	0	1.81	401.64
*Franchise sum	7170	92.67	32.03	0	69.74	95.15	117.17	318.21
Medicine for Acute disease	7170	201.99	116.74	0	121	181	260	1300
Medicine for chronic disease	7170	295.92	332.40	1	90	187	375	3937
*Income	7170	5338.16	1006.31	2620.54	4700.47	5150.92	5615.74	10175.47

* Every cost is expressed in US dollars (USD)

Appendix table S2 K-means method descriptive statistics for the third cluster of the low risk class								
Features	Nmber of insured	Mean	Std	Min	%25	%50	%75	Max
*Insurance paid sum	19419	205.05	73.92	0.40	152.27	212.34	266.40	320.86
age	19419	21.99	17.46	6	13	18	23	101
Medicine sum	19419	322.67	282.88	0	147	262	408	3883
Prescription sum	19419	61.23	24.84	1	44	60	78	170
*Sum of Insurance paid & Deductions	19419	208.02	75.16	0.40	154.15	215.20	269.89	491.24
*Deductions _sum	19419	2.975406	8.51	-17.46	0	0	1.57	193.49
*Franchise sum	19419	85.59	34.74	0	61.09	87.76	111.72	350.48
Medicine for Acute disease	19419	180.22	122.20	0	85	162	250	2060
Medicine for chronic disease	19419	130.32	234.80	0	20	56	136	3463
*Income	19419	3715.44	2995.46	1290.11	1570.10	2476.77	4659.04	12382.2

* Every cost is expressed in US dollars (USD)

Appendix tableS3 K-means method descriptive statistics for the second cluster of the middle risk class								
Features	Number of insured	Mean	Std	Min	%25	%50	%75	Max
*Insurance paid sum	23321	617.17	187.11	320.88	454.93	599.60	768.82	990.89
age	23321	52.60	7.33	27	48	52	56	99
Medicine sum	23321	1364.85	1562.50	86	678	1059	1723	116147
Prescription sum	23321	142.16	50.89	21	104	135	174	400
*Sum of Insurance paid & Deductions	23321	624.906	190.58	320.88	460.52	606.65	778.06	1803.49
*Deductions _sum	23321	7.73	25.02	-95.02	0	0.44	5.78	1189.9
*Franchise sum	23321	256.63	86.36	0	187.01	248.73	319.88	1829.42
Medicine for Acute disease	23321	354.65	229.61	0	199	306	455	2650
Medicine for chronic disease	23321	959.30	1518.47	3	328	643	1253	115819
*Income	23321	5383.35	1111.38	1290.11	4700.47	5150.92	5615.74	10175.47

* Every cost is expressed in US dollars (USD)

Appendix table S4 K-means method descriptive statistics for the third cluster of the middle risk class								
Features	Number of insured	Mean	Std	Min	%25	%50	%75	Max
*Insurance paid sum	25107	546.04	172.77	320.87	402.85	504.67	659.96	991.00
age	25107	25.99	23.82	5	12	18	25	101
Medicine sum	25107	820.82	898.79	7	290	544	960	30311
Prescription sum	25107	132.98	48.42	4	98	125	160	412
*Sum of Insurance paid & Deductions	25107	552.18	175.16	311.51	407.25	510.21	667.50	1201.17
*Deductions _sum	25107	6.14	16.59	-65.75	0	0.60	5.03	556.70
*Franchise sum	25107	226.16	81.02	0	166.24	209.62	273.08	3390.21
Medicine for Acute disease	25107	302.52	229.18	0	132	255	409	5462
Medicine for chronic disease	25107	483.59	783.67	2	65	175	504	8754
*Income	25107	3505.24	2789.66	1290.11	1570.10	2029.40	4325.58	12382.27

* Every cost is expressed in US dollars (USD)

Appendix table S5 K-means method descriptive statistics for the second cluster of the high risk class								
Features	Number of insured	Mean	Std	Min	%25	%50	%75	Max
*Insurance paid sum	28504	2446.92	4519.96	991.03	1191.95	1506.2	2115.62	129627.7
age	28504	49.53	17.96	6	40	48	59	101
Medicine sum	28504	5093.16	159654	18	1677	2771.5	4547	16826624
Prescription sum	28504	303.12	130.88	3	217	279	361	1324
*Sum of Insurance paid & Deductions	28504	2467.33	4534.91	926.593	1204.83	1520.07	2141.72	129631.1
*Deductions _sum	28504	20.41	114.13	-610.69	0.22	2.867898	14.01	10576.02
*Franchise sum	28504	784.74	1009.26	0	479.63	599.65	814.17	38386.06
Medicine for Acute disease	28504	743.17	5886.94	0	370	596	910.25	990774
Medicine for chronic disease	28504	2622.80	3238.22	0	1025	1974	3603	245201
*Income	28504	7485.72	1878.11	1950.51	6861.83	7670.81	8987.65	10175.47

* Every cost is expressed in US dollars (USD)

Appendix table S6 K-means method descriptive statistics for the third cluster of the high risk class								
Features	Number of insured	Mean	Std	Min	%25	%50	%75	Max
*Insurance paid sum	5847	2976.12	6277.03	991.10	1154.16	1456.11	2328.26	169486.6
age	5847	47.89	31.73	5	18	36	82	101
Medicine sum	5847	3163.80	2892.35	34	1056.5	2460	4594.5	87296
Prescription sum	5847	259.60	121.90	3	178	241	316	1166
*Sum of Insurance paid & Deductions	5847	2994.39	6294.01	939.56	1166.86	1471.43	2346.31	169491.3
*Deductions _sum	5847	18.27	79.60	-121.28	0.06	2.50	12.37	3450.113
*Franchise sum	5847	818.16	1080.38	0	454.80	560.75	784.79	30485.52
Medicine for Acute disease	5847	645.17	581.568	0	233	483	876	6756
Medicine for chronic disease	5847	2429.39	2523.36	8	494.5	1679	3700.5	60152
*Income	5847	3852.55	2353.38	1290.11	1885.26	3463.18	4537.25	12382.27

* Every cost is expressed in US dollars (USD)
